# Implications of climate and land-use change for landscape processes, biodiversity, ecosystem services, and governance

**DOI:** 10.1007/s13280-014-0596-6

**Published:** 2015-01-09

**Authors:** Bodil Elmhagen, Ove Eriksson, Regina Lindborg

**Affiliations:** 1 Department of Zoology, Stockholm University, 106 91 Stockholm, Sweden; 2 Department of Ecology, Environment and Plant Sciences, Stockholm University, 106 91 Stockholm, Sweden; 3 Department of Physical Geography and Quaternary Geology, Stockholm University, 106 91 Stockholm, Sweden

**Keywords:** Interdisciplinary research, Drivers of change, Spatiotemporal scales, Feedback processes, Complex interactions

## Abstract

This introduction to the Special Issue summarizes the results of 14 scientific articles from the interdisciplinary research program Ekoklim at Stockholm University, Sweden. In this program, we investigate effects of changing climate and land use on landscape processes, biodiversity, and ecosystem services, and analyze issues related to adaptive governance in the face of climate and land-use change. We not only have a research focus on the 22 650 km^2^ Norrström catchment surrounding lake Mälaren in south-central Sweden, but we also conduct research in other Swedish regions. The articles presented here show complex interactions between multiple drivers of change, as well as feedback processes at different spatiotemporal scales. Thus, the Ekoklim program highlights and deals with issues relevant for the future challenges society will face when land-use change interacts with climate change.

## Introduction

Human population growth and the associated increase in resource use have been major drivers of global change since around the year 1800, causing the species’ extinction rate to rise markedly above the background level (Millennium Ecosystem Assessment 2005; Steffen et al. 2007). In recent decades, climate change and consequent impacts on biodiversity and human societies have received much political and scientific interest. However, while climate change is projected to become the second most important driver of global biodiversity change in the twenty-first century, land use is projected to remain the most important driver (Sala et al. 2000). Biodiversity loss changes the structure and function of ecosystems, and this can in turn affect human societies through changes in ecosystem services delivery (Cardinale et al. 2012), i.e., ecosystem properties that societies require and make use of (Fisher et al. 2009).

To meet such challenges, research is needed to understand the interplay between multiple drivers, biodiversity, and ecosystem services at landscape to regional scales (Foley et al. 2011). Under the scientific program Ekoklim at Stockholm University, we have developed an interdisciplinary research environment focusing on the intersection between climate, land use, water use, biodiversity, and social–ecological studies at different spatiotemporal scales (www.zoologi.su.se/ekoklim/). The main objective of this collaboration is to generate new insights for improved management and governance of ecosystem services in dynamic landscapes.

**Fig. 1:**
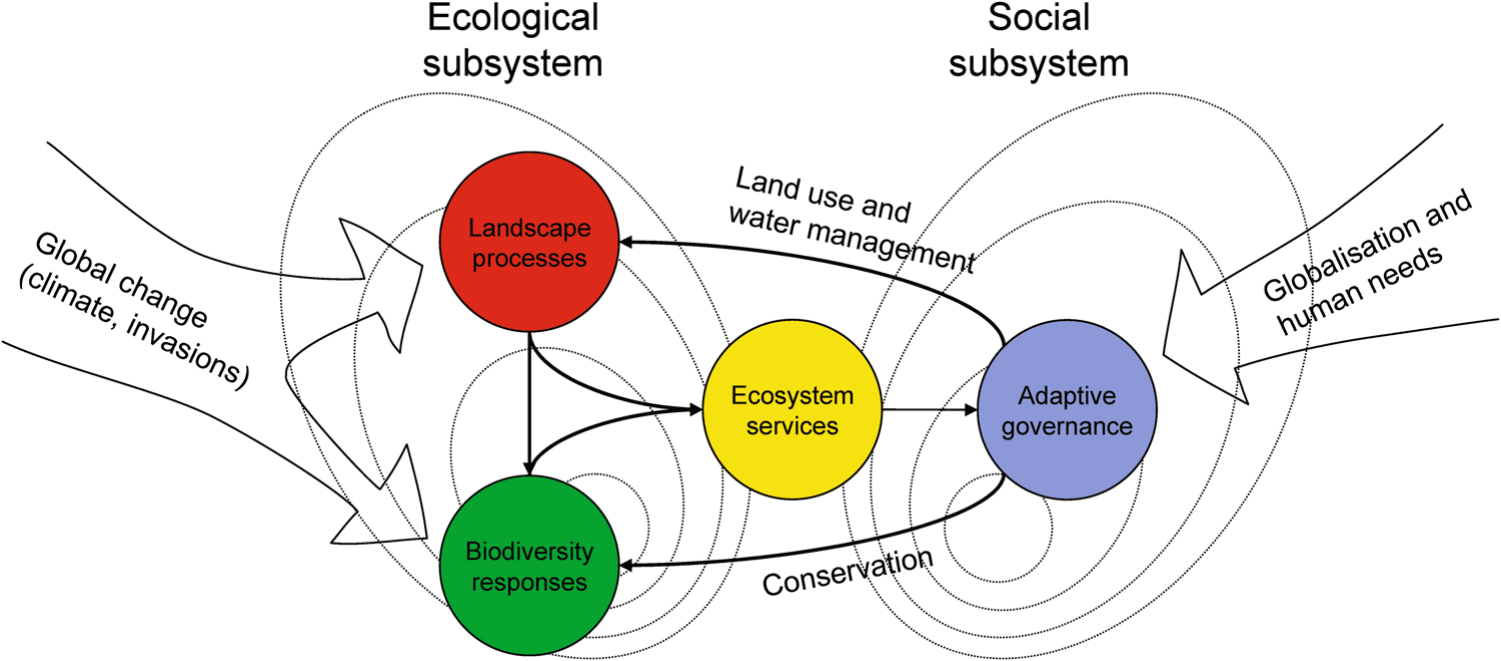
Conceptual model of the scientific themes which are the bases for the Ekoklim research program and this Special Issue

In this Special Issue, we present 14 scientific articles based on results from Ekoklim. The research is structured around four closely interacting clusters: landscape processes, biodiversity responses, ecosystem services, and adaptive governance (Fig. 1).

A rationale for the structure of the Ekoklim program, and a basic premise of the research, is that the complexities in how climate and land-use change affect ecological and social systems, as well as the complexities for society to handle and mitigate effects of these changes, necessitate research collaboration among scientists from multiple disciplines. There are several recent examples of such complexities dealt with by researchers from the Ekoklim program. Land-use change is often associated with water-use change, which in turn can have feedback effects on water circulation in the landscape as well as on the local and regional climates (Destouni et al. 2013). In the biotic environment, species can respond to change either through evolution, adapting to new conditions, or by tracking suitable conditions through dispersal. However, species with high dispersal rates may actually increase large-scale biodiversity loss in response to climate change as they may become invasive and drive other species to extinction through changed species interactions (Norberg et al. 2012). Furthermore, the impact of climate change on biodiversity should differ depending on the status of certain species in the ecosystem, which carry out regulating top-down ecosystem functions, while the strength of top-down effects can be modified by land use (Ripple et al. 2014). The effect of management actions aiming to preserve biodiversity can also vary due to local climate and biotic conditions (Sletvold et al. 2013). Finally, climate change effects on human societies will depend on their response strategies, which in turn rely on the quality of available information and the capacity to make informed decisions (Boyd et al. 2013).

## Summary of articles in this Special Issue

### Landscape processes

To understand the effects of climate change on biodiversity and human society, as well as the responses of society, the scientific community must take into account both climate-related changes in the abiotic environment, effects of other drivers such as land use, and potential interactions and synergies between drivers. Three papers explore different aspects of these issues. Verrot and Destouni (2015) assess changes in soil moisture in response to climate change over 60 years in two Swedish drainage basins. Soil moisture is the amount of water in the unsaturated zone between the land surface and the groundwater table, which affects and is affected by hydroclimatic as well as ecological conditions. Verrot and Destouni show that the long-term average and intra-annual variation in soil moisture have been stable over time, whereas the between-year variation has increased, suggesting an increase in extreme climate events. Cousins et al. (2015) explore regional land-use change over the last century in a 1652 km^2^ area in south-eastern Sweden. The amount of seminatural grasslands in the landscape has decreased from 46 to 2 %, primarily transitioned to silviculture, i.e., managed forests dominated by conifers. Forests are generally associated with low biodiversity, while there are small hotspots of biodiversity in seminatural grasslands and habitats that have transitioned from seminatural grasslands to wetlands or broadleaf forests. This study reveals a pervasive redistribution of biodiversity at the landscape scale, as well as substantial declines in biodiversity outside remaining biodiversity hotspots. Strandmark et al. (2015) describe a number of potential effects that climate change might have on ecosystems in the Baltic Sea borderland between land and sea. These coastal ecosystems will experience sea level rise as well as changes in salinity and a decrease in winter ice cover, but coastal areas are also highly exploited. The borderland ecosystems are therefore likely to experience coastal squeeze between sea and settlement, which may prevent migration of animal and plants in response to sea level rise. To handle such complex problems, it is necessary to improve communication among multiple actors, including authorities, scientists, NGOs, and other representatives of the general public.

### Biodiversity responses

One of the most generally acknowledged effects of climate change relates to species geographic ranges. Elmhagen et al. (2015) synthesize the state-of-the-art knowledge about range shifts and trends in abundance of birds and mammals in the Scandinavian border zone between boreal forest and tundra. They find evidence suggesting that many species have become affected by climate change during the last century. Southern species have expanded northward, whereas northern species have declined, partly as a result of interactions with the expanding southerners. They conclude that climate and land-use change likely had synergistic effects on the community. These combined effects of climate and land use are also stressed by Auffret et al. (2015) who examine the relationship between connectivity and ecological resilience, i.e., how the ability of individuals to move between habitat patches in the landscape affects species persistence. Auffret et al. particularly highlight the importance of temporal connectivity, i.e., persistence in the same habitat patch over time. Temporal connectivity in refugia can also act as an insurance against environmental variation and prolong the persistence of a species. For example, plants have evolved bet-hedging strategies such as perenniation, clonality, and persistent seeds, which increase temporal connectivity locally, but also regionally, as refugia populations can serve as sources from which individuals disperse to new locations. Hylander et al. (2015) explore the phenomenon of microrefugia, small areas where a species may survive in an otherwise hostile region, as important components of species’ response to climate change. There are conditions that are necessary for microrefugia to develop, suggesting that in order to benefit from microrefugia, species should be limited by climatic factors that are decoupled from the regional climate.

Another direct effect of climate change concerns phenology, i.e., the temporal manifestation of biological features such as leaf burst and flowering in plants, development of life cycles in insects, or arrival of migrating birds. Based on a unique phenological dataset, Kullberg et al. (2015) show how spring arrival in migratory birds has changed across Scandinavia by comparing spring arrivals during 1873–1917 and the present time. One of their key results is that short-distance migrants have been more affected by climate change than long-distance migrants. Surprisingly, this effect is not consistent across latitudes, as it disappears when moving northward. Navarro-Cano et al. (2015) investigate phenological effects in more detail focusing on interactions between species, an often neglected aspect of phenology. If interactions between species are altered because interacting species respond differently to climate change, then this may potentially have more drastic implications for ecological systems than range shifts. Navarro-Cano et al. examine this by studying the interactions between a common butterfly, the Orange Tip Butterfly *Antocharis cardamines*, and its host plants. This study illustrates the complexities in the response of even a single species to climate change, thus suggesting that predictions are uncertain.

### Ecosystem services

One of the main goals of the Ekoklim program is to use an ecosystem service approach to assess benefits from different ecosystems and landscape processes. Quieroz et al. (2015) combine GIS data from the Norrström catchment with publicly available data to study synergies and trade-off among ecosystem services across 62 municipalities. They find five distinct bundles of ecosystem services, i.e., ecosystem services spatially agglomerated in the landscape, which could be explained by regional social and ecological gradients. These bundle groups are, for example, “mosaic cropland-horses,” “mosaic cropland-livestock,” and “forest and towns.” They also show that human-dominated landscapes are highly multifunctional, e.g., urban areas were hotspots of cultural services. Information on such bundles and trade-offs between ecosystem services can be an important tool for governance when planning services at the municipal level. At the same time, sustainable landscape management also needs to understand processes occurring at smaller, local-to-landscape scales, especially with regard to ecosystem service supply and demand. Andersson et al. (2015) compare two contrasting Swedish farming systems (low intensity and high intensity) through a set of landscape indicators using existing in situ qualitative and quantitative data. The quantity of most ecosystem services differed between farming systems as did the ways the farmers viewed them. The relationships between indicators addressing the same service are often complex, and supply and demand can be linked to both the social and physical sides of ecosystem service generation. This complexity pinpoints the importance of understanding services as integrated social–ecological processes and that qualitative information can inform quantitative measures to better plan and manage rural landscapes.

Wetlands are often highlighted as important providers of multiple ecosystem services, the sustainable use of which requires knowledge of the underlying ecological mechanisms. Functional trait-based approaches and particularly the community-weighted mean trait (CWMT) provide a strong link between species communities and ecosystem functioning. Moor et al. (2015) combine species distribution modeling and plant functional traits to estimate the direction of change of ecosystem processes under climate change in three main wetland types in the Norrström drainage basin. They show that species compositional changes tend to increase CWMT values of specific leaf area and canopy height, whereas changes in root depth vary with wetland type, leading to a proportional shift toward faster growing, more productive, and taller species. In terms of ecosystem service provision, this suggests a potential increase in flood attenuation services, a potential increase in short-term (but not long-term) nutrient retention, and ambiguous outcomes for carbon sequestration.

Quin et al. (2015) show that the potential of nutrient retention in wetlands also depends on large-scale patterns of water-flow in the landscape. By developing a general analytic model, Quin et al. quantify the nutrient retention contribution of wetlands for multiple sub-catchments in two Swedish Water Management Districts. They find that the retention contribution of wetlands and other landscape features is significant only if a large fraction of the total waterborne pollutant transport passes through them. This means that there are no detectable effects of wetlands on the landscape-scale retention of nutrients from, for example, agricultural sources, although the total nutrient retention is correlated with the transport distance to the sea. These results emphasize the need for informed consideration of the large-scale pathway distributions of water flow and pollutant transport through catchments to accurately understand and quantify the large-scale ecosystem service of water, retention of pollutants and nutrients. While land-use changes can alter wetland services, knowledge of processes and limitations to the vegetation’s potential to deliver services may help in strategic and adaptive planning, for example, where to restore or create wetlands, and of what type and size, in response to land use elsewhere in the catchment.

### Adaptive governance

Two contributions directly deal with problems related to governance under conditions of climate and land-use change. Kininmonth et al. (2015) focus on collaboration among managing actors, using a novel network approach to analyze how 25 municipalities in central Sweden coordinate wetland management. Since the distribution of natural resources is not necessarily congruent with administrative boundaries, efficient management depends critically on coordination of governance. Their results suggest that coordination in this case was satisfactory, although coordination was perhaps not intentional in the first place, and often (unintentional or not) reliant upon a set of intermediate municipalities acting as coordinators. The network approach provides an important tool to analyze the capacity of society to manage common boundary-spanning resources.

Boyd et al. (2015) have a broader objective, dealing with “anticipatory governance,” a concept that has become in focus particularly in the face of climatic uncertainty. The first section of their paper contains a review of “anticipation,” which is an often-used, but less well-defined, term in environmental social sciences, and such a review is therefore much needed. Boyd et al. then continue to examine how anticipatory governance is manifested in relation to water management in the Ekoklim target catchment area Norrström. Boyd et al. illustrate how an anticipatory approach can inform adaptive institutions, decision-making, strategy formation, and societal resilience.
